# Diversification in *Salmonella* Typhimurium DT104

**DOI:** 10.3201/eid1106.050100

**Published:** 2005-06

**Authors:** John Threlfall, Katie L. Hopkins, Linda R. Ward

**Affiliations:** * Health Protection Agency, Centre for Infections, London, United Kingdom

**Keywords:** Salmonella, molecular epidemiology, Antibiotic resistance, multi-drug resistance

**To the Editor:** Multidrug-resistant (MDR) *Salmonella* Typhimurium definitive phage type (DT) 104 with chromosomally encoded resistance to ampicillin, chloramphenicol, streptomycin/spectinomycin, sulfonamides, and tetracyclines (ACSSpSuT) was first identified and characterized in the United Kingdom in the early 1990s (1). MDR DT104 has subsequently caused numerous outbreaks throughout the world (1). The organism is characterized by a distinctive *Xba* I-generated pulsed-field profile (PFP), designated Xtm 1 (2), carriage of the 90-kb *S*. Typhimurium serovar-specific plasmid (SSP), and presence of the 43-kb *Salmonella* genomic island 1 (SGI 1), which is composed of integrons containing, respectively, the ASu (*bla*_CARB-2_ and *sul1*) and SSp (*aadA2*) genes, with intervening plasmid-derived genes coding for chloramphenicol/florfenicol (*cmlA*) and tetracycline resistance (*tetG*) (1,3,4). The same genetic characteristics have been observed in MDR strains of the closely related DTs 12 and 104b (5) and in some strains of phage type U302 (4). All isolates of MDR DT104 ACSSpSuT contain the same gene cassettes irrespective of source or country of origin. Although MDR DT104 has declined during the last 5 years, the organism remains the most common MDR *Salmonella* in the United Kingdom and many other European countries (6).

Since 1998, MDR DT104 has undergone changes in both resistance spectrum and genetic structure. In the United Kingdom, outbreaks of MDR DT104 have been caused by new subclones with additional resistance to trimethoprim (Tm) (R-type ACSSpSuTTm) (7), by clones with decreased susceptibility to ciprofloxacin (Cp_L_) (R-type ACSSpSuTCp_L_) (1), and by strains of R-type SSpSu. In 2002, an outbreak of MDR DT104 ACSSpSuTTm with >200 cases was recognized (7). The outbreak strain was characterized by 3 plasmids of 6.8, 3.0, and 1.5 kb. The 6.8-kb plasmid coded for resistance to sulfonamides and trimethoprim, with trimethoprim resistance being mediated by *dhfr1b*. The outbreak strain lacked the *S*. Typhimurium SSP and was negative by polymerase chain reaction (PCR) for a 437-bp internal fragment of the *Salmonella* plasmid virulence (*spv*)*C* gene. The absence of the SSP was reflected in the PFP, which was identical to Xtm 1 but lacked a fragment of ≈90 kb that corresponds to the presence of the SSP (4). A strain of R-type ACSSpSuT that also lacked the SSP and with a PFP indistinguishable from that of the ACSSpSuTTm strain caused a simultaneous outbreak with >40 cases (7). This strain was also characterized by 2 plasmids of 3.0 and 1.5 kb but did not possess the 6.8-kb sulfonamide-trimethoprim resistance plasmid.

Decreased susceptibility to ciprofloxacin coupled with resistance to nalidixic acid (Nx) was first reported in MDR DT104 in 1996 in the United Kingdom (1). Four mutations in *gyrA*, each giving rise to resistance to Nx/Cp_L_, were subsequently identified in MDR DT104. The most common mutation was aspartate (Asp)-87 to asparagine (AAC) and involved a change from aspartate (GAC) to AAC. An identical mutation was identified in a MDR DT104 strain responsible for an outbreak in Denmark in 1998 (8). In 1999, an Asp-87 to glycine mutation was identified in a Cp_L_ MDR DT104 strain responsible for a major outbreak in northwestern England (1). Subsequently, >500 sporadic MDR DT104 infections in the United Kingdom have involved strains with Cp_L_.

In 2004, 2 simultaneous outbreaks of DT104 were recorded in the United Kingdom in which the strains were of R-type SSpSu, 1 involving >100 cases and 1 involving >50 cases. Isolates were characterized by pulsed-field gel electrophoresis coupled with plasmid profile analysis. Resistance genes were identified by using PCR with primers specific for *bla*_TEM_ (A), *bla*_CARB-2_, *cmlA*, *catI* (C), *catIII* (C), *aadA2*, *sul1*, and *tetG* (4). Testing for SGI1 was conducted by using primers U7-L12 and LJ-R1 for the left junction and primers 104-RJ and 104-D for the right junction (9). All isolates exhibited the Xtm 1 PFP, but strains from the 2 outbreaks could be differentiated by the presence or absence of an additional plasmid of ≈3 kb. By using PCR, all isolates possessed *aadA2* and *sul1* but were negative for *bla*_TEM_,*bla*_CARB-2_, *cmlA*, *catI*, *catIII*, and *tet*G. Similarly, all strains were positive for the left junction of SGI1. These results indicate that the SGI1 was in the same chromosomal location as for DT104 ACSSpSuT but was lacking the right junction.

These findings demonstrate that evolutionary changes have occurred involving both loss and acquisition of drug resistance genes, the development of chromosomal mutations conferring resistance to Nx/Cp_L_, and in certain subclones, the loss of the SSP. None of these changes has altered the virulence of the organism, with bloodstream invasion in only 1.6% of 408 cases in the United Kingdom. These findings contrast with those in the United States, where MDR nontyphoidal *Salmonella* have been associated with excessive numbers of bloodstream infections (10).

All facets of the organism should be explored for epidemiologic investigations. These should include phage type, antibiogram, plasmid profile, and PFP, coupled with the identification of resistance genes and integrons, and, when appropriate, the characterization of mutations conferring resistance to quinolone antimicrobial agents.

These studies were in part supported by a grant from the Department for Environment, Foods and Rural Affairs, reference V02136.
